# Supplementary material for: Association between oral microbiome and seven types of cancers in East Asian population: a two-sample Mendelian randomization analysis
Source: Front Mol Biosci. 2023 Nov 21;10:1327893. doi: 10.3389/fmolb.2023.1327893 (PMC10702768; doi:10.3389/fmolb.2023.1327893)
Supplement: Supplementary file 1 [file DataSheet1.ZIP › Supplementary Materials/MR plots for tongue/tongue═╝/Ovarian cancer/pheno.965_to_ovarian cancer_leave_one_out.pdf]

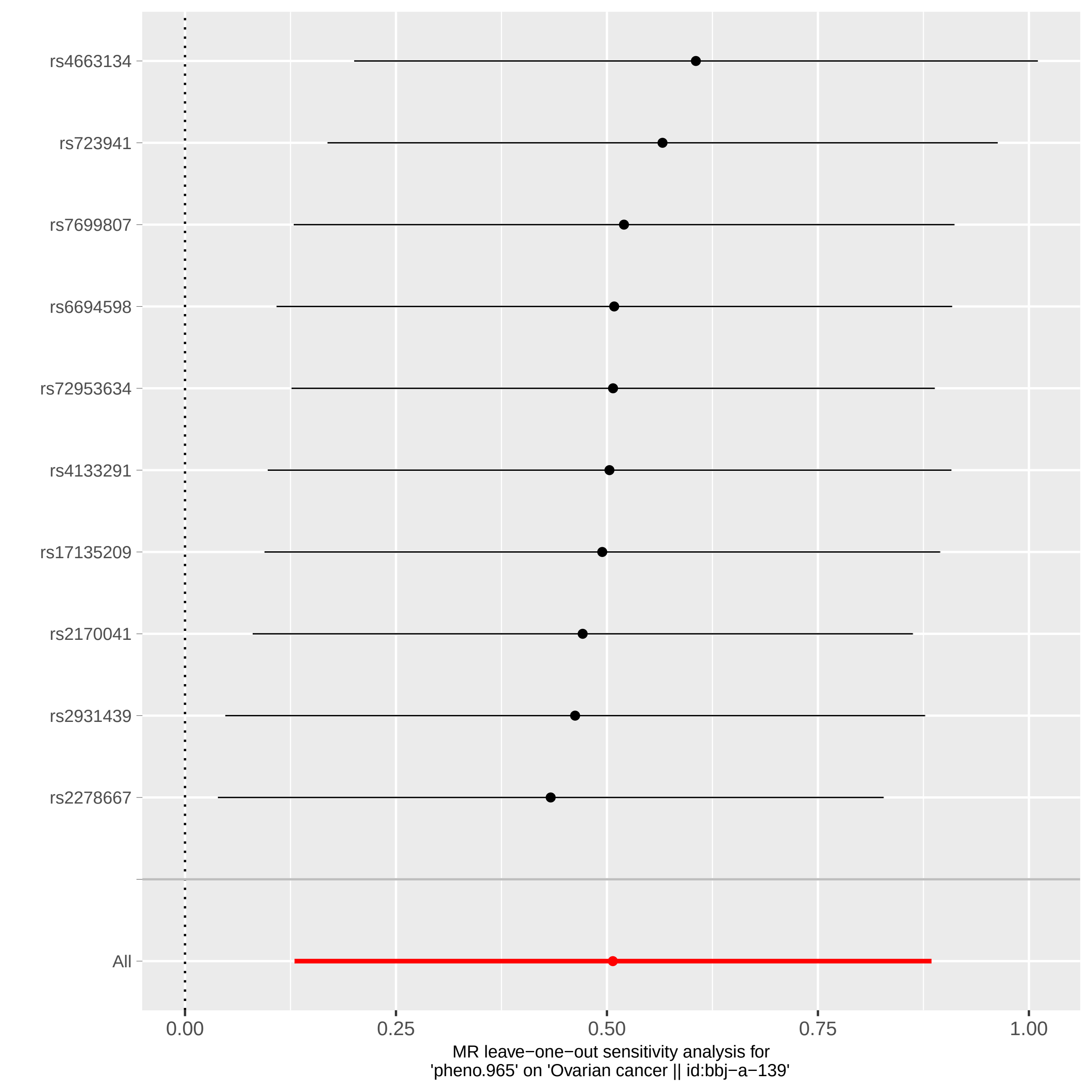
rs4663134
rs723941
rs7699807
rs6694598
rs72953634
rs4133291
rs17135209
rs2170041
rs2931439
rs2278667
All
0.00
0.25
0.50
0.75
1.00
MR leave−one−out sensitivity analysis for
'pheno.965' on 'Ovarian cancer || id:bbj−a−139'
